# Cementless Transtrochanteric Bipolar Hemiarthroplasty vs. Proximal Femoral Nailing for Unstable Intertrochanteric Fractures in the Elderly: A Retrospective Comparative Study

**DOI:** 10.3390/jcm15010151

**Published:** 2025-12-25

**Authors:** Yusuf Polat, Tolga Keçeci, Murat Alparslan, Abdullah Alper Şahin, Alper Çıraklı, Serkan Sipahioğlu

**Affiliations:** Department of Orthopaedics, Ordu University Training and Research Hospital, 52200 Ordu, Turkey; tolgakececil@odu.edu.tr (T.K.); muratalparslan61@gmail.com (M.A.); abdullahalpersahin@odu.edu.tr (A.A.Ş.); alperomu@gmail.com (A.Ç.); serkansipahioglu@odu.edu.tr (S.S.)

**Keywords:** proximal femoral nail, bipolar hemiarthroplasty, unstable intertrochanteric fractures

## Abstract

**Background/Objectives**: Unstable intertrochanteric femur fractures (IFFs) in geriatric patients are associated with high rates of morbidity and mortality due to poor bone quality, multiple comorbidities, and limited functional capacity. This study aimed to compare the clinical outcomes of cementless bipolar hemiarthroplasty (BHA) performed via a transtrochanteric approach and proximal femoral nailing (PFN) in elderly patients with unstable IFFs. **Methods**: This retrospective comparative study included 131 patients aged ≥70 years who underwent surgery for AO/OTA 31-A2 and 31-A3 unstable fractures between January 2021 and July 2025 were retrospectively reviewed. 64 patients received cementless BHA and 67 underwent PFN. Eligible patients were ambulatory prior to fracture (independently or with a cane/walker); patients with pathological fractures/malignancy, alternative procedures (cemented or posterolateral BHA, total hip arthroplasty, tumor prosthesis, or other osteosynthesis methods), incomplete records, or <6 months of follow-up were excluded. Demographics, perioperative variables, mechanical complications, revision requirement, time to mobilization, and 1- and 6-month mortality rates were analyzed. Primary outcomes were mortality and perioperative clinical parameters. **Results**: The two groups were comparable in age, sex, ASA scores, and fracture patterns. Intraoperative blood loss and transfusion requirements were significantly higher in the BHA group (both *p* < 0.001). Mobilization was observed earlier in patients treated with BHA (1 [1,2] vs. 3 [2,3] days; *p* < 0.001). Mechanical complications were more frequently observed after PFN, which was associated with a higher revision requirement (17.9% vs. 4.7%; *p* = 0.018). Operative time, hospital stay, and 1- and 6-month mortality rates showed no significant differences between the groups. **Conclusions**: In geriatric patients with unstable IFFs, cementless BHA performed via a transtrochanteric approach may be considered a viable surgical option with appropriate patient selection, taking into account its association with earlier mobilization and the observed mechanical complication profile. PFN offers advantages of reduced blood loss and lower transfusion needs. Surgical decision-making should be individualized based on fracture morphology, bone quality, and the patient’s overall medical condition. Given the heterogeneity of unstable fractures within the AO/OTA classification and the retrospective nature of the present study, larger, multicenter prospective investigations incorporating functional outcomes are warranted to further clarify optimal treatment strategies.

## 1. Introduction

The rapid increase in the elderly population has led to a substantial rise in intertrochanteric femur fractures (IFFs), which now represent a major public health challenge affecting both clinical outcomes and health-care economics [1]. In geriatric patients, poor bone quality, multiple comorbidities, and limited functional capacity substantially increase the risk of complications, particularly in unstable fracture patterns. In cases where adequate fracture stability cannot be achieved, prolonged immobilization inevitably leads to significant functional decline, increased morbidity, and a high mortality rate [2].

Although proximal femoral nailing (PFN) is widely preferred in the treatment of intertrochanteric femur fractures today, the risk of mechanical complications increases markedly in geriatric patients due to poor bone quality and comminuted fracture morphology. Consequently, the rates of implant failure, malunion, and nonunion become substantially higher [3,4,5]. Achieving adequate reduction and maintaining stable fixation are often challenging in AO/OTA 31A2–A3 fracture patterns, particularly when posteromedial support is deficient and accompanied by lateral wall compromise [6]. These biomechanical limitations may compromise the effectiveness of load-sharing osteosynthesis techniques in elderly patients and have prompted increasing interest in arthroplasty-based strategies for selected unstable fracture patterns.

In this context, bipolar hemiarthroplasty (BHA) emerges as an important treatment option for patients with limited compliance to postoperative mobilization, relatively short life expectancy, and inadequate fracture stability [7,8]. Although it carries risks such as increased blood loss, infection, and dislocation, the ability to allow immediate full weight-bearing—thereby allowing earlier functional recovery and potentially decreasing the need for caregiving, which has been reported as a potential advantage of BHA [9,10,11]. In addition, the preservation of posterior soft tissues through a transtrochanteric approach may contribute to reduced blood loss and a lower risk of dislocation [12,13]. The development of cementless, distally fixed modular stem designs is considered a significant technical advancement, as it enables the prevention of cement-related cardiopulmonary complications while providing early stable fixation [14].

A substantial portion of the existing literature has focused on comparing internal fixation with BHA or on evaluating different surgical approaches used during arthroplasty. However, studies directly comparing specifically between PFN and cementless BHA performed via a transtrochanteric approach in elderly patients with unstable IFFs are remarkably limited. Therefore, the aim of our study is to compare these two surgical methods in terms of perioperative parameters, mechanical complications, the need for revision, early mobilization, and short-term mortality. Our hypothesis is that BHA is associated with earlier mobilization and a lower requirement for revision surgery compared with PFN.

## 2. Materials and Methods

### 2.1. Study Design and Ethical Approval

This retrospective comparative study includes patients who underwent surgery for unstable IFF’s between January 2021 and July 2025. The study protocol was approved by the Ordu University Non-interventional Clinical Research Ethics Committee (Date: 7 November 2025, Approval No: 2025/372).

### 2.2. Patient Selection

**Figure 1:**
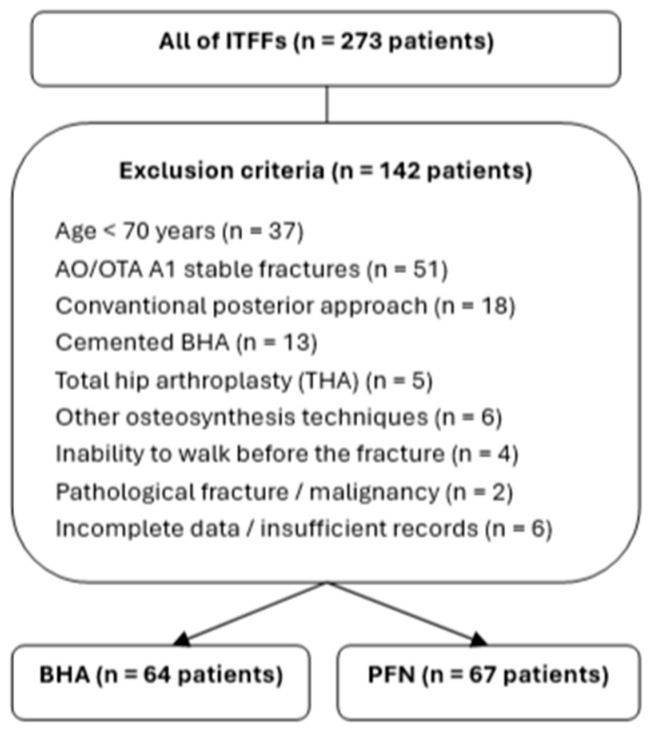
Flowchart of patient selection.

A total of 131 patients aged ≥70 years who were treated with either cementless BHA using a transtrochanteric approach or PFN and who were able to ambulate independently or with a cane/walker prior to the fracture were included. Patients who underwent cemented BHA or BHA through a conventional posterolateral approach; those treated with total hip arthroplasty, tumor prosthesis, or alternative osteosynthesis methods; and individuals with pathological fractures or malignancy were excluded. Patients with incomplete imaging or operative records and those with less than six months of follow-up were also excluded (Figure 1).

All patients were evaluated in terms of age, sex, fracture side, AO/OTA classification, ASA score, operative time, intraoperative blood loss, transfusion requirement, need for revision, and postoperative complications. First- and six-month mortality data were obtained retrospectively from the hospital’s digital medical records.

AO/OTA A2 and A3 fracture patterns were considered unstable and included in the study, whereas A1 types—representing stable fracture patterns—were excluded from the analysis. The surgical method was determined based on the fracture morphology, bone quality, and the patient’s overall clinical condition, in accordance with the surgeon’s experience and preference. The decision was made as part of routine clinical care, before the study was conceived or designed, and was not guided by any specific algorithm or classification-based protocol. All surgeries were performed by orthopedic surgeons experienced in hip fracture management.

### 2.3. Radiological Assessment

Preoperative evaluation, early postoperative assessment, and follow-up visits were performed using standard anteroposterior and lateral hip radiographs in all patients. Fracture type was determined on preoperative radiographs according to the AO/OTA classification. All radiological assessments were independently reviewed by two orthopedic surgeons experienced in hip fracture surgery, and disagreements were resolved through consensus. Follow-up radiographs were systematically examined for fixation integrity, implant position, and any potential mechanical changes. In the PFN group, implant position, cephalic screw placement, and quality of reduction were evaluated, whereas in the BHA group, femoral stem alignment, joint congruency, and healing of the greater trochanter fragments (cortical continuity and callus formation) were assessed.

### 2.4. Definition of Complications

**Figure 2:**
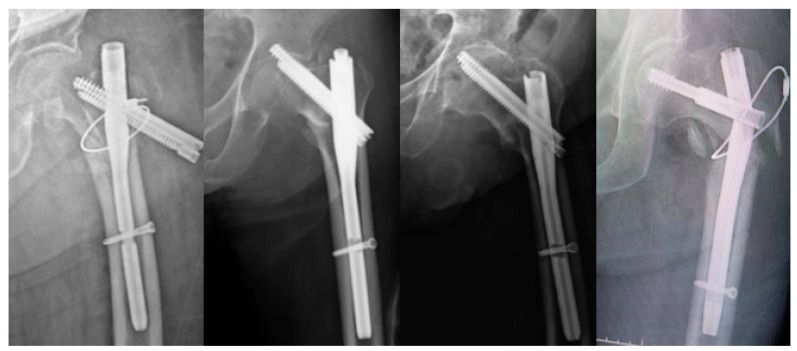
Postoperative complications after PFN for unstable IFFs.

Dislocation in BHA patients was diagnosed clinically and confirmed radiographically. Trochanteric nonunion in the BHA group was defined as proximal migration of the greater trochanter fragment due to absent or insufficient healing (fibrous union) and was considered a mechanical complication resulting in fixation failure. In the PFN group, mechanical complications were defined as conditions leading to loss of stability, including superior migration of the cephalic screw with acetabular penetration or loss of fixation (cut-out), medial advancement of the screw toward the center of the femoral head (cut-through), backing-out of the lag screw, significant loss of reduction (varus collapse, malunion), nonunion, and implant breakage (Figure 2). In the BHA group, mechanical complications were defined as implant-related events leading to loss of stability, including dislocation and periprosthetic fracture. Revision-requiring mechanical complications were defined as implant-related mechanical failures necessitating secondary surgical intervention.

Superficial infection was defined as involvement limited to the skin and subcutaneous tissues requiring antibiotic treatment only, whereas deep infection was defined as an infection involving the joint or prosthesis that required surgical debridement, with or without implant removal.

**Figure 3:**
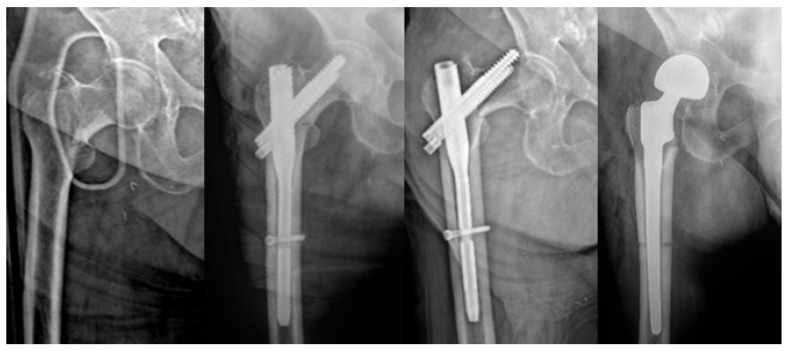
Early postoperative cut-out after PFN and subsequent revision with cementless hemiarthroplasty.

Revision was defined as any reoperation indicated due to loss of implant stability, development of mechanical complications, or deep infection (Figure 3). However, revision surgery could not be performed in all patients despite being indicated. The decision to proceed with revision was based on the patient’s overall condition, comorbidities, anesthesia risk, and the risk-benefit balance of additional surgical intervention. Accordingly, revision requirement refers to cases in which revision surgery was clinically indicated, regardless of whether revision was actually performed.

### 2.5. Surgical Technique

#### 2.5.1. Bipolar Hemiarthroplasty

**Figure 4:**
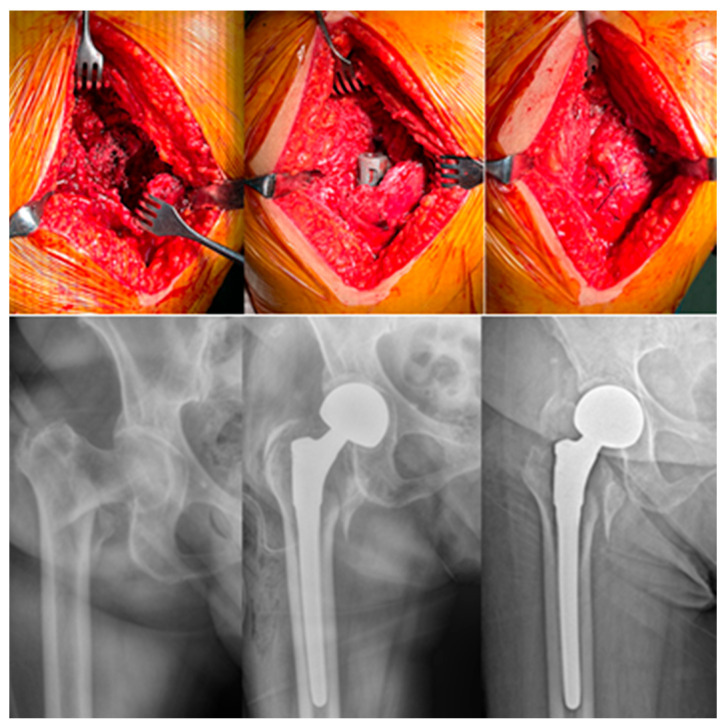
Cementless modular bipolar hemiarthroplasty via a transtrochanteric approach.

The patient was positioned in the lateral decubitus position. Following a posterolateral skin incision, the tensor fascia lata was incised, and the gluteus maximus fibers were split. The approach was carried out between the trochanteric fracture fragments while preserving the short external rotators. By retracting the gluteus medius and the attached greater trochanter fragments, the femoral neck fracture line and femoral head were exposed. A 2 cm incision was made in the superior portion of the capsule, and the femoral head was removed using a corkscrew extractor. The medullary canal was prepared and a cementless femoral stem was inserted. Appropriate modular neck and bipolar head components were selected, and the joint was reduced. The capsule was repaired primarily. Fracture fragments of the greater trochanter were fixed using heavy nonabsorbable sutures, a cerclage cable, or a trochanteric grip plate, depending on fracture pattern and fragment stability. After placement of a negative-pressure drain, the soft tissues were closed anatomically, completing the procedure (Figure 4).

#### 2.5.2. Proximal Femoral Nail

The patient was positioned supine. Fracture reduction was attempted using a closed technique under fluoroscopic guidance; open reduction was performed in cases where an adequate reduction could not be achieved. After reduction, the entry point was identified over the apex of the greater trochanter, and a guidewire was advanced into the intramedullary canal. The canal was prepared with an appropriately sized reamer, and a nail of suitable length was inserted. Proximal locking screws (the lag screw and the antirotation screw) were then placed under fluoroscopic control. Distal locking screws were inserted using either the freehand technique or a targeting device. After confirming the final implant positions and fracture reduction with fluoroscopy, the soft tissue layers were closed anatomically, completing the procedure.

### 2.6. Postoperative Mobilization and Rehabilitation Protocol

Prior to the fracture, all patients were able to ambulate independently or with the assistance of a cane or walker. A standardized rehabilitation protocol, implemented by the same physiotherapy team, was used for both groups postoperatively. All patients were encouraged on postoperative day 1 to sit at the edge of the bed and stand with assistance as tolerated.

In the BHA group, full weight-bearing with a walker was initiated as tolerated, starting on postoperative day 1, provided that the patient’s general condition and vital parameters were stable. In the PFN group, the weight-bearing protocol was individualized based on the surgeon’s intraoperative assessment of stability, fracture morphology, and the patient’s overall clinical status. While some patients were permitted early partial weight-bearing, those with insufficient stability followed a more gradual progression. This approach reflects real-life clinical practice and resulted in controlled variability in mobilization timing.

### 2.7. Statistical Analysis

All statistical analyses were performed using SPSS version 24.0 (IBM Corp., Armonk, NY, USA). The distribution characteristics of continuous variables were assessed with the Shapiro-Wilk test.

Variables with normal distribution were presented as mean ± standard deviation (mean ± SD) and compared between groups using the independent samples *t*-test. Non-normally distributed variables were expressed as median (minimum-maximum or IQR) and analyzed using the Mann-Whitney *U* test.

Categorical variables were reported as counts and percentages (%). Group differences were evaluated using the chi-square test or Fisher’s exact test when expected cell frequencies were insufficient. A *p*-value < 0.05 was considered statistically significant for all analyses.

## 3. Results

A total of 131 patients were included in the study; 64 underwent cementless BHA via a transtrochanteric approach, and 67 were treated with PFN. The demographic and clinical characteristics of the groups are presented in Table 1. The mean age was 83.3 ± 6.2 years in the BHA group and 81.5 ± 6.6 years in the PFN group, with no significant difference between the two (*p* = 0.122). The groups were also comparable in terms of sex distribution (*p* = 0.160), fracture side (*p* = 0.798), ASA scores (*p* = 0.151), and AO/OTA fracture patterns (*p* = 0.654).

Perioperative variables are presented in Table 2. There was no significant difference between the BHA and PFN groups in terms of operative time (55 [50–65] min vs. 60 [50–70] min; *p* = 0.398). Intraoperative blood loss was significantly higher in the BHA group (350 [250–400] mL vs. 150 [150–200] mL; *p* < 0.001). Similarly, the need for blood transfusion was markedly greater in the BHA group (2 [1–3] vs. 1 [0–1]; *p* < 0.001). The time to initiation of mobilization was significantly shorter in the BHA group (1 [1,2] days), enabling much earlier mobilization compared with the PFN group (3 [2,3] days) (*p* < 0.001). Postoperative hospital stay, however, was similar between the groups (*p* = 0.697).

The distribution of complications is presented in Table 3. There were no statistically significant differences between the groups in terms of superficial infection (4 vs. 2; *p* = 0.319) or deep infection (3 vs. 1; *p* = 0.292). In the BHA group, dislocation was observed in 1 patient and trochanteric nonunion in 9 patients. In the PFN group, nonunion was detected in 3 patients, malunion in 11 patients, and implant failure in 15 patients.

The need for revision was markedly higher in the PFN group (12 patients, 17.9%), and this difference was statistically significant (χ^2^ = 5.65, *p* = 0.018). The number of patients who actually underwent revision surgery was 3 (4.7%) in the BHA group and 7 (10.4%) in the PFN group, with no significant difference between the groups (*p* = 0.215).

The 1-month mortality rate was 7.8% (*n* = 5) in the BHA group and 3.0% (*n* = 2) in the PFN group, with no significant difference (*p* = 0.267). At 6 months, mortality was 7.8% (*n* = 5) in the BHA group and 13.4% (*n* = 9) in the PFN group; similarly, no significant difference was observed between the groups (χ^2^ = 1.08, *p* = 0.298). Although no statistically significant difference was observed, the numerically higher 6-month mortality rate was noted in the PFN group compared with the BHA group.

## 4. Discussion

Our study compared cementless BHA performed via a transtrochanteric approach with PFN in geriatric patients with unstable intertrochanteric femur fractures and demonstrated notable clinical differences between the two surgical techniques. Our findings indicate that BHA was associated with earlier mobilization and a lower need for revision. Although PFN offers certain perioperative benefits, such as reduced blood loss and decreased transfusion requirements, it exhibited a higher tendency for mechanical failure due to the biomechanical characteristics of unstable fracture patterns

Early mobilization is a critical factor in the management of geriatric patients with unstable IFFs, as prolonged immobilization is associated with increased morbidity and potentially life-threatening complications [15,16,17]. In the present study, patients treated with cementless BHA were mobilized earlier than those treated with PFN, a finding that is consistent with previous reports emphasizing the potential benefits of immediate or early weight-bearing after arthroplasty. In contrast, elderly patients treated with PFN may experience delayed recovery, as limited upper-extremity muscle strength can make mobilization with assistive devices challenging when full weight-bearing is restricted [7,18]. However, mobilization timing may have been influenced by postoperative weight-bearing protocols. Nevertheless, these protocols are related to the stability provided by the surgical technique in routine clinical practice; therefore, the earlier mobilization observed in the hemiarthroplasty group should be interpreted not as an isolated advantage of the surgical technique, but as the result of a combined effect of surgical strategy and postoperative management.

The two techniques differed markedly in terms of the need for revision. In the BHA group, revision was required only in cases of deep infection, including a single episode of prosthetic dislocation. In contrast, most revisions in the PFN group were attributable to mechanical complications. Implant failure (cut-out, cut-through, back-out), nonunion, and malunion—complications that compromise biomechanical stability—constituted the primary reasons for revision in the PFN group. This finding is consistent with previous studies demonstrating that the biomechanical characteristics of unstable fractures increase the risk of failure in osteosynthesis procedures and supports the notion that PFN is more prone to mechanical failure, particularly in unstable fracture types [19,20].

At this point, it is important to emphasize that conversion arthroplasty performed after failed internal fixation entails substantial challenges. Compared with primary hemiarthroplasty, conversion procedures are more complex and technically demanding due to implant removal, altered anatomy, and associated bone loss [21]. It is also associated with higher complication rates and poorer clinical outcomes compared with primary surgery [22,23].

When evaluating the impact of the surgical approach, the transtrochanteric approach has been associated with lower reported dislocation rates in the literature. It is well established that capsular and short external rotator release in the posterolateral approach represents a significant risk factor for dislocation [24]. In contrast, the transtrochanteric approach described by Bombacı for intertrochanteric fractures has been shown to significantly reduce dislocation rates by preserving the posterior soft tissues, as demonstrated by both Gülsoy et al. and Gökalp et al. [13,25]. The observation of only a single dislocation in our series—markedly lower than the rates reported in posterolateral BHA series involving geriatric patients—further supports the advantage of the transtrochanteric approach.

Implant selection in arthroplasty is also of particular importance in the geriatric population. In cemented arthroplasties performed for proximal femur fractures, a significant increase in intraoperative mortality has been reported, which may be associated with increased embolic load, hypovolemia, and the limited cardiopulmonary reserve of frail elderly patients [26]. However, considering that metaphyseal press-fit stability may not always be sufficient in the osteoporotic proximal femur, the use of modular stems capable of providing secure fixation at the diaphyseal or isthmus level has become increasingly widespread [14,27]. In our study, the use of cementless modular stems allowed early weight-bearing while maintaining intraoperative hemodynamic stability, representing a potentially favorable option for the geriatric population.

Our findings regarding trochanteric nonunion are consistent with the literature. Although the nine cases of nonunion observed in our series may pose concerns in terms of abductor mechanism weakness, limping, and an increased risk of dislocation, it is well recognized that fibrous union is often clinically tolerable in elderly patients with low functional demand [28,29]. Because trochanteric nonunion did not lead to a need for revision surgery in our patient cohort, it was not considered an implant-related mechanical failure and was therefore not included in the mechanical failure analysis.

The absence of a notable difference in operative time between the two techniques is noteworthy. PFN, being a minimally invasive method, has generally been associated with shorter operative durations [15]. However, in unstable fractures, achieving an acceptable reduction may require additional manipulation, auxiliary techniques, and increased fluoroscopy use, all of which can prolong the operative time. Therefore, the variability in surgical duration is likely related primarily to the morphological characteristics of the fracture and the extent of intervention required by the surgical technique [30].

The lower perioperative blood loss observed in the PFN group is an expected finding and is consistent with the physiological advantages of a minimally invasive technique [31,32]. Consistent with the literature, this difference did not appear to have a significant adverse impact on mortality, infection rates, or systemic complications [33].

In terms of infection, although the number of deep infections was higher in the BHA group, this difference was not statistically significant. However, the broader soft-tissue dissection required in BHA is clinically important, as it may increase susceptibility to infection in geriatric patients, particularly those with advanced age and multiple comorbidities.

Regarding hospital stay, despite the early mobilization advantage of BHA, the postoperative length of stay was similar between the groups. This finding likely reflects the fact that discharge decisions in elderly patients depend not only on mobility but also on several other factors, including transfusion requirements, early postoperative infection, and the management of comorbidities.

Finally, the similar mortality rates observed between the groups indicate that survival in the elderly fracture population is determined more by patient-specific factors than by the surgical technique itself. The literature widely supports the notion that factors such as advanced age, comorbidity burden, frailty profile, and preoperative functional status play a more decisive role in mortality outcomes [10,30,34]. Our findings suggest that the choice of surgical technique does not play a decisive role in short-term mortality.

The choice between PFN and BHA in clinical practice is based on an integrated assessment of patient frailty, bone quality, and the expected ability to comply with postoperative partial weight-bearing restrictions. BHA may be considered in patients with severe osteoporosis or a low likelihood of adhering to partial weight-bearing protocols to facilitate early mobilization and reduce the risk of fixation failure, whereas PFN may be more appropriate in patients with better bone quality, particularly when perioperative blood loss represents a greater clinical concern. Importantly, the lower revision requirement observed with BHA does not justify its routine use but supports individualized, risk-benefit-based decision-making in selected patients. Surgical preferences may also vary according to the surgeon’s experience and treatment philosophy [35].

This study has several limitations. First, due to its retrospective design, it was not possible to completely eliminate observer bias during data collection and evaluation. Second, the absence of a predefined algorithm or objective set of criteria guiding the selection of surgical technique may have introduced potential selection bias. Unmeasured or uncontrolled baseline differences between groups may have existed, limiting the ability to draw definitive causal inferences from the observed outcomes. Third, because the maximum follow-up period was limited to four years, long-term complications such as aseptic loosening, protrusio acetabuli, late infection, or late dislocation could not be assessed. Fourthly, clinical parameters essential in geriatric fracture care, such as functional outcomes, pain levels, walking independence, and discharge destination, as well as common geriatric complications including delirium, pneumonia, deep vein thrombosis, and pulmonary embolism, were beyond the scope of this study’s analysis. Furthermore, since postoperative weight-bearing protocols were not standardized and were determined retrospectively, the observed differences in mobilization timing should be interpreted with caution. Moreover, potential variations in the PFN technique, including differences in nail type and screw positioning, were not separately analyzed, and this may have influenced the outcomes. Finally, this study reflects the experience of a single center, which may limit the external validity and generalizability of the findings to other clinical settings.

## 5. Conclusions

In conclusion, our findings underscore the importance of individualizing surgical decision-making for unstable IFFs by considering fracture morphology, bone quality, and the patient’s overall clinical condition. Cementless BHA performed via a transtrochanteric approach may be considered a viable surgical option in selected geriatric patients, given its association with earlier mobilization and the observed mechanical complication profile. PFN, on the other hand, offers advantages related to lower blood loss and reduced transfusion requirements. Given the heterogeneity of unstable fractures within the AO/OTA classification and the retrospective nature of the present study, larger, multicenter prospective investigations incorporating functional outcomes are warranted to further clarify optimal treatment strategies.

## Figures and Tables

**Table 1:** Demographic and Clinical Characteristics.

Variables	BHA	PFN	Statistics	*p* Value
Age (mean ± SD)	83.3 ± 6.2	81.5 ± 6.6	t = 1.56	0.122
Sex (M/F)	21/43	30/37	χ^2^ = 1.971	0.160
Side (Left/Right)	32/32	32/35	χ^2^ = 0.066	0.798
ASA score			χ^2^ = 3.78	0.151
II	4 (%6)	11 (%16)		
III	40 (%63)	34 (%51)		
IV	20 (%31)	22 (%33)		
AO classification			χ^2^ = 3.30	0.654
31A2.1	24 (%37)	24 (%36)		
31A2.2	14 (%22)	15 (%22)		
31A2.3	17 (%27)	20 (%30)		
31A3.1	1 (%2)	3 (%4)		
31A3.2	2 (%3)	0 (%0)		
31A3.3	6 (%9)	5 (% 8)		

Continuous variables are presented as mean ± SD; categorical variables as *n* (%). *p*-values were calculated using the independent samples *t*-test for normally distributed variables, the Mann-Whitney *U* test for non-normally distributed variables, and the chi-square test for categorical variables.

**Table 2:** Perioperative variables.

Variables	BHA	PFN	Statistics	*p* Value
Operative time (min)	55 (50–65)	60 (50–70)	*U* = 1820.5	0.398
Intraoperative blood loss (mL)	350 (250–400)	150 (150–200)	*U* = 310.0	<0.001
Transfusion requirement	2 (1–3)	1 (0–1)	*U* = 837.5	<0.001
Mobilization (days)	1 (1–2)	3 (2–3)	*U* = 801.0	<0.001
Postoperative hospital stay (days)	4 (4–5)	4 (3–5)	*U* = 2062.0	0.697

Data are presented as median (IQR). Group comparisons were performed using the Mann-Whitney *U* test.

**Table 3:** Postoperative complications.

Variables	BHA *n* (%)	PFN *n* (%)	Statistics	*p* Value
Superficial infection	4 (%6)	2 (%3)		0.319 ^a^
Deep infection	3 (%4)	1 (%1)		0.292 ^a^
Dislocation	1 (%1)	—		—
Trochanteric nonunion	9 (%14)	—		—
Nonunion	—	3 (%4)		—
Malunion	—	11 (%16)		—
Implant failure	—	15 (%22)		—
Revision requirement	3 (%4)	12 (%18)	χ^2^ = 5.65	0.018 ^b^
Revision surgery	3 (%4)	7 (%10)		0.215 ^a^
1-month mortality	5 (%7)	2 (%3)		0.267 ^a^
6-month mortality	5 (%7)	9 (%13)	χ^2^ = 1.08	0.298 ^b^

Categorical variables are presented as *n*. ^a^ Using Fisher probabilities in 2 × 2 table. ^b^ Using chi-squared test with Yates’ correction. χ^2^: chi-squared test. —: not available.

## Data Availability

The datasets generated and analyzed during the current study are available from the corresponding author upon reasonable request.

## Abbreviations

The following abbreviations are used in this manuscript:

IFF	Intertrochanteric femur fracture
BHA	Bipolar hemiarthroplasty
PFN	Proximal femoral nail
AO/OTA	Arbeitsgemeinschaft für Osteosynthesefragen/Orthopaedic Trauma Association
ASA	American Society of Anesthesiologists
